# Is cardiopulmonary transit time (CPTT) measured by using dynamic rubidium cardiac PET/CT a predictor for cardiac function?

**DOI:** 10.1007/s10554-025-03346-5

**Published:** 2025-02-14

**Authors:** Lena C. Seige, Boya Zhang, Jakob Heimer, Noel Spielhofer, Cristina Popescu, Karsten Murray, Christian La Fougère, Irene A. Burger, Alexander W. Sauter

**Affiliations:** 1 Department of Nuclear Medicine, Cantonal Hospital Baden, Partner Hospital for Research and Teaching of the Medical Faculty of the University of Zurich, Baden, 5404 Switzerland; 2 University Hospital Basel, University of Basel, Basel, 4031 Switzerland; 3 Department of Mathematics, Seminar for Statistics, ETH Zurich, Zurich, 8092 Switzerland; 4 Department of Cardiology, Cantonal Hospital Baden, Baden, Baden, 5404 Switzerland; 5 Department of Nuclear Medicine and Clinical Molecular Imaging, University Hospital Tuebingen, 72074 Tuebingen, Germany; 6 Department of Nuclear Medicine, University Hospital Zurich, University of Zurich, Zurich, 8006 Switzerland; 7 Department of Radiology, University Hospital Tuebingen, 72076 Tuebingen, Germany

**Keywords:** Positron Emission Tomography Computed Tomography, Rubidium-82, Myocardial perfusion imaging, Echocardiography, Pulmonary transit Time

## Abstract

Cardiopulmonary transit time (CPTT) represents the time needed for the circulation of blood from the right to the left ventricle. This parameter can be measured during dynamic acquisition of rubidium ([^82^Rb]) cardiac PET/CT. To further characterize this marker, we wanted to assess the association between CPTT and parameters of cardiac function derived from echocardiography. Retrospective single center analysis of patients referred to [^82^Rb]RbCl-PET/CT with rest/stress protocol on an integrated hybrid PET/CT system (Biograph mCT, Siemens Healthineers, Erlangen, Germany) and echocardiography within 100 days. After intravenous injection of 7.5 MBq/kg [^82^Rb]RbCl dynamic scans with initially 12 × 10 s frames were started. For data analysis a volume of interest (VOI) was drawn in the left and right ventricle using dedicated software. The difference between the peak time for the two time activity curves (TAC) was extracted as CPTT and normalized for heart rate (NCPTT). Associations between NCPTT and echo parameters such as left ventricular ejection fraction (EF_Echo_) were analyzed using linear regression models. 44 patients (sex: 28 male, 16 female) were enrolled with a time difference between PET and echocardiography of 19.65 ± 23.3 days. 9 patients had a rest CPTT of 0 s, 32 patients 10 s and 3 patients 20 s. The association between EF_Echo_ and NCPTT revealed a significant negative correlation (beta = -0.77; CI: -1.32, -0.22; *p* = 0.007). Given this association, univariate predictive models for EF_Echo_ were applied. Root mean square error was 6.83% for the EF_PET_, and 6.0% for NCPTT, which indicates a slightly higher predictive performance for the NCPTT model with a lower error. Pulmonary transit time can be estimated with [^82^Rb]RbCl-PET/CT, with a high positive association to rest EF_Echo_. However, smaller time frames than 10 s are needed, for more accurate estimation of cardiac function.

**Graphical Abstract:**

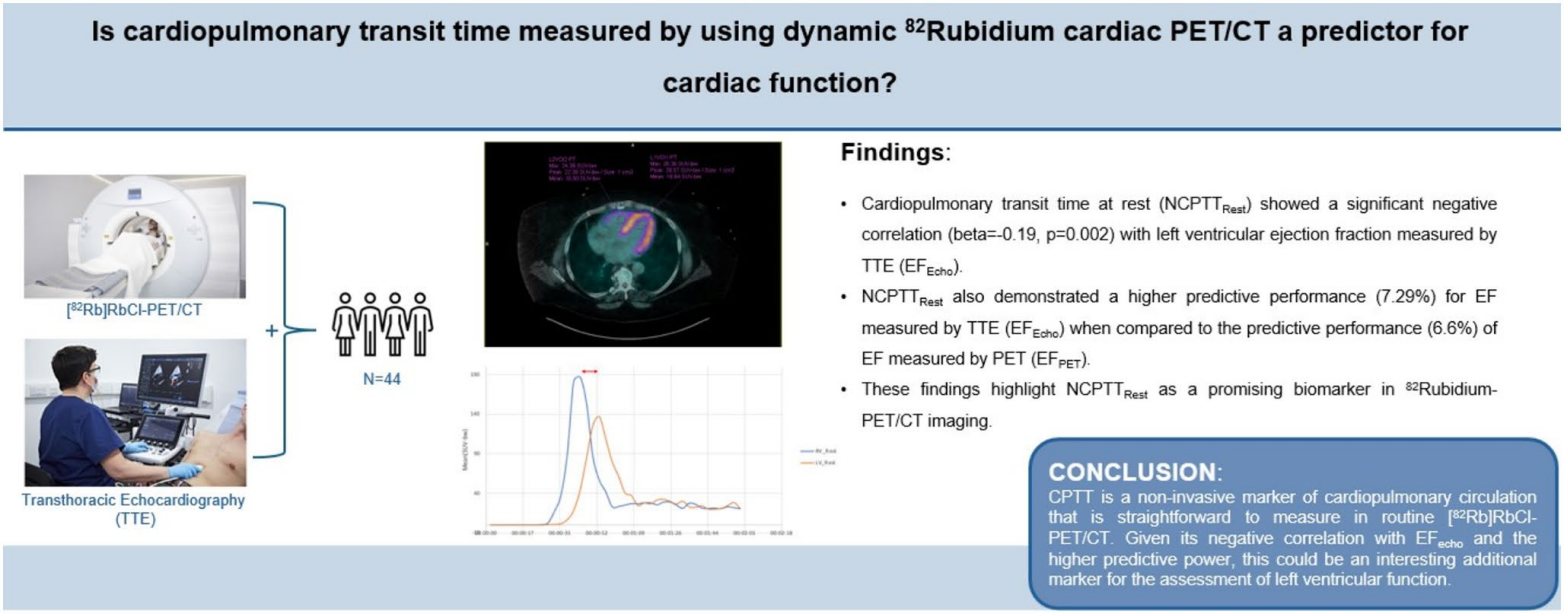

## Introduction

Myocardial perfusion imaging (MPI) using positron emission tomography (PET) is an advanced, non-invasive technique that enhances the capabilities of conventional MPI. This technology allows for precise quantification of myocardial blood flow (MBF) during rest and stress conditions, as well as the measurement of myocardial flow reserve (MFR). Recent studies have demonstrated that impaired MFR, as assessed by PET, is a significant predictor of adverse cardiovascular events [1]. Additionally, PET imaging is instrumental in detecting various stages of coronary artery disease (CAD), ranging from early atherosclerosis and coronary microvascular dysfunction (CMD) to advanced, flow-limiting epicardial disease. As part of the Rubidium82-cardiac-PET/CT examination ([^82^Rb]RbCl-PET/CT), left ventricular ejection fraction (LVEF) is assessed both at rest and under stress conditions. However, previous studies have indicated that PET- or SPECT-derived EF is prone to error and underestimation [2, 3]. Therefore, identifying a more robust biomarker during [^82^Rb]RbCl-PET/CT that correlates better with cardiac function would be beneficial. Identifying such parameters could help determine whether additional assessment with TTE is necessary for a more comprehensive evaluation of cardiac function. Numerous studies have identified significant correlations between cardiopulmonary transit time (CPTT), measured using cardiac MRI, and key indicators of diastolic and systolic cardiac function. However, only a few have explored the potential associations between [^82^Rb]RbCl-PET/CT-derived CPTT and cardiac function parameters [4–7].

Therefore, in this study we sought to investigate the clinical feasibility and value of CPTT determination derived from [^82^Rb]RbCl-PET/CT. We did this by exploring possible associations between CPTT and echocardiographic parameters. We hypothesized that CPTT is significantly correlated with these echocardiographic parameters, potentially enhancing our understanding of cardiac function. Echocardiography as a reference was chosen due to its widespread use and availability.

## Materials and methods

### Study design and population

**Fig. 1:**
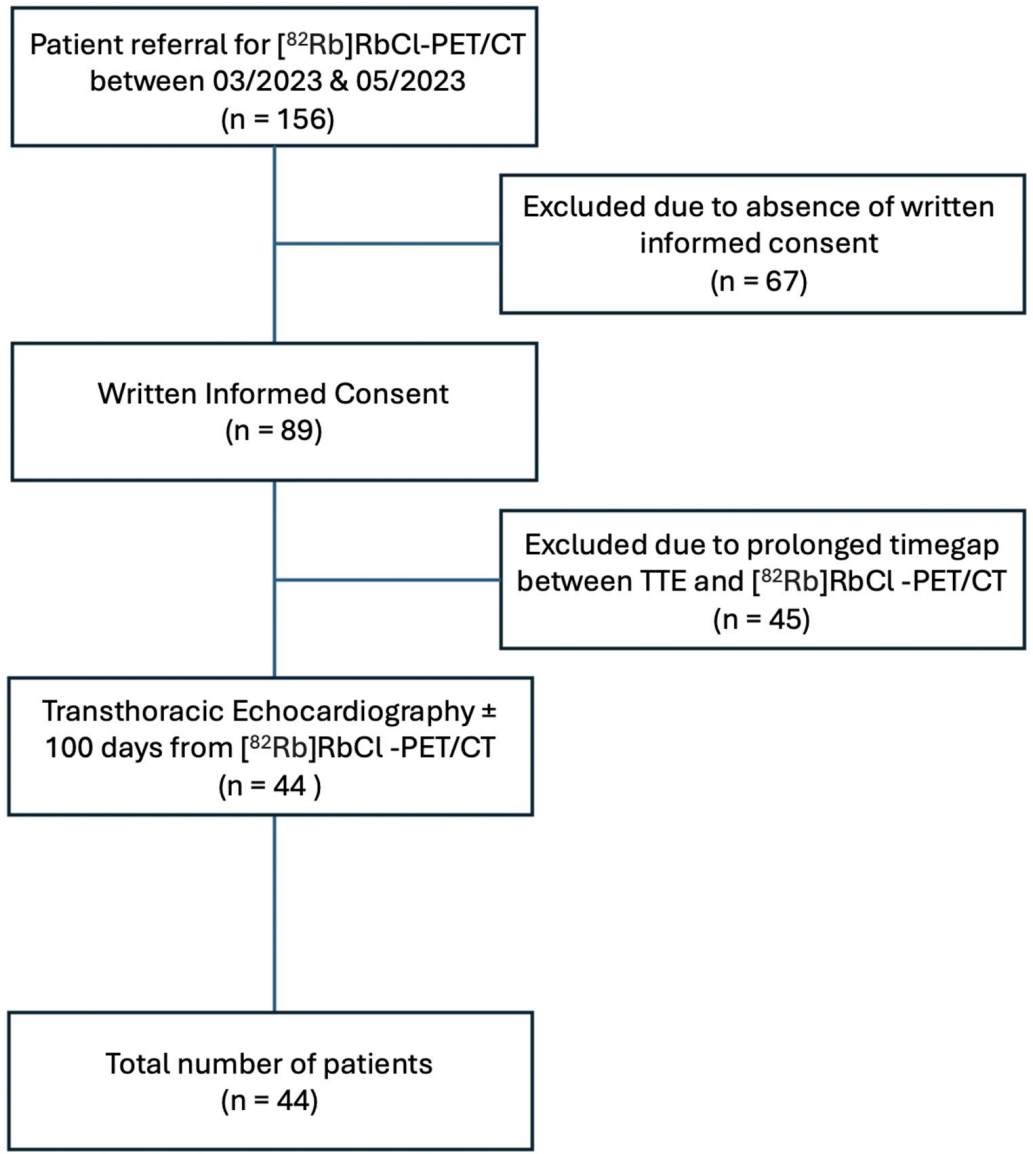
Study flow chart

This was a retrospective study of patients referred for [^82^Rb]RbCl-PET/CT, between February 2023 and May 2023. [^82^Rb]RbCl-PET/CT is a non-invasive technique for the assessment of myocardial perfusion in patients at risk for coronary artery disease. Forty-four patients (mean age: 72.4 ± 11.3 years) were included from the outpatient clinic of a single center (Department of Nuclear Medicine, Kantonsspital Baden, Switzerland). In addition, patient records were screened for TTE within 100 days before or after [^82^Rb]RbCl-PET/CT. The study flow chart is shown in Fig. 1. Exclusion criteria for the patients included: (i) Pregnancy or breastfeeding, (ii) Food intake within four hours before [^82^Rb]RbCl-PET/CT, (iii) intake of caffeine/teein within twelve hours before [^82^Rb]RbCl-PET/CT. The study was conducted in accordance with ICH-GCP guidelines and the Declaration of Helsinki. Written informed consent was obtained from all patients and the protocol was approved by the northwest and central Switzerland ethics committee (2023–02188).

### Imaging protocols

#### PET acquisition

[^82^Rb]RbCl-PET/CT was performed using a Siemens Biograph mCT Flow (Siemens Healthineers, Munich, Germany). The injected dose of Rubidium-Chloride ([^82^Rb]RbCl) was 7.5 MBq/kg. The maximum injected dose was not more than 1125 MBq (mean 595 ± 151.9 MBq). Among other sequences, dynamic images were reconstructed with the following framesets according to vendor-supplied defaults: 1 s delay, 12 × 10 s, 2 × 30 s, 2 × 60 s, 1 × 120 s. Reconstruction parameters: reconstruction method OSEM3D + TOF 3i21s, scatter correction method, model-based, absolute scatter scaling, matrix. In addition, immediately before starting the PET Scan, a low-dose CT-scan was performed for attenuation correction and calcium scoring according to the EANM guidelines [8]. [^82^Rb]RbCl-infusions were performed at the maximum flow rate of 30 mL/min, to achieve the shortest possible bolus infusion. All scans were initiated manually after [^82^Rb]RbCl-infusion was started, and the scanner-reported coincidence (prompt) count rates exceeded 10 kcps. For each patient rest and consecutive stress imaging was performed. Pharmacological stress was achieved using adenosine. Adenosine was injected for 6 min at a dose of 0.14 mg/kg/min. After 2 min and 45 s adenosine infusion, the injection with Rubidium-Chloride was started (Rubyscan, Jubilant). Contraindications for adenosine administration include chronic obstructive pulmonary disease (COPD) and asthma [8]. In cases of contraindication a single injection of regadenoson (Rapiscan^®^,400mcg/5 ml) was administered.

#### Image analysis

**Fig. 2:**
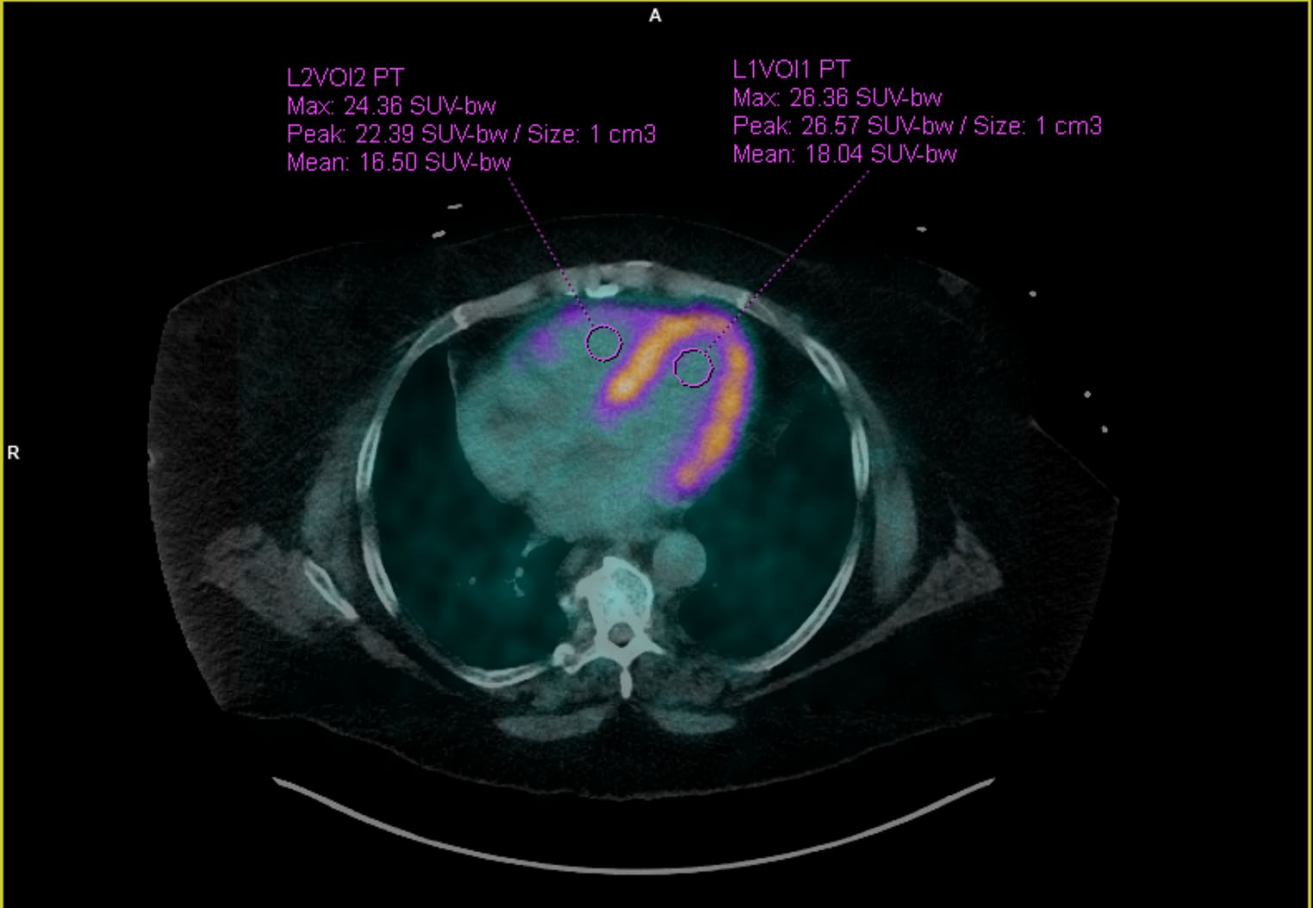
PET activity over time is obtained from the left and right ventricular cavities shown in the axial view. VOIs are placed in the left (L1VOI1) and the right (L2VOI2) ventricular cavities

**Fig. 3:**
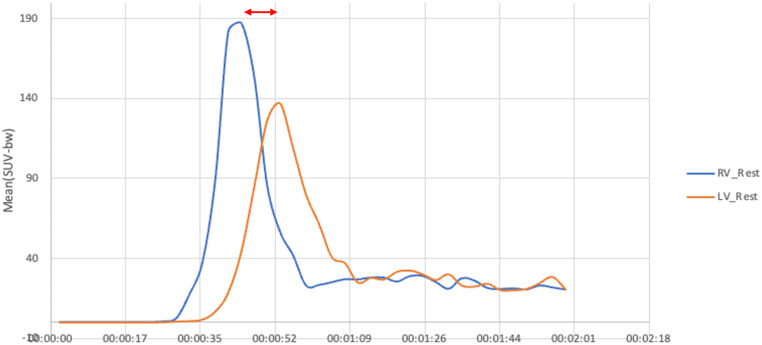
Time activity curves during rest first pass perfusion: right ventricular cavity (blue) and left ventricular cavity (orange). Peak to peak measurement (red line) to isolate the CPTT, demonstrating the time the bolus of injected activity (SUV_mean_) needed to get from the right to the left ventricle

Image analysis was performed centrally by a MD student (B.Z.) using standardized 1 cm³ volumes of interest (VOI) placed in the left and right ventricular cavities in the horizontal long-axis view, using syngo.via (MM Oncology, version VB60A, Siemens Healthineers) and double checked by a dual board certified nuclear medicine physician and radiologist (A.W.S.) (Fig. 2). Correct, intracavital position was assured using early dynamic PET data and CT correlation. Time activity curves of mean standardized uptake values (SUV_mean_) were obtained. These curves featured a sharp peak of activity, indicating the arrival of the bolus of injected activity in each cavity during first pass perfusion. For each activity curve, the peak was isolated and used to measure the mean arrival time of the bolus (SUV_mean_) with the difference between these times representing the CPTT (Fig. 3).

### Transthoracic Echocardiography (TTE)

Two-dimensional and Doppler Transthoracic Echocardiography were performed within 100 days before or after [^82^Rb]RbCl-PET/CT examination. The subjects were examined in the left-lateral decubitus position using a Vivid E9 commercial ultrasound scanner (version BT11, General Electric Healthcare, Horten, Norway) with phased-array transducers (M5S-D and 4 V-D). Two-dimensional data acquisitions included parasternal long and short-axis views, and three standard apical views. For each view, two to three consecutive cardiac cycles were recorded during quiet respiration. From the apical four- and two-chamber views, left ventricular (LV) volumes were measured according to Simpson’s rule. These echocardiographic examinations were conducted by various board-certified cardiologists, both internal and external to our institution. The cardiologists measured and computed several parameters, including LV ejection fraction (EF_Echo_), mitral valve inflow pattern (E and A velocity; E/A ratio) and the tricuspid annular plane systolic excursion (TAPSE).

### Statistical analysis

All statistical analyses were conducted with R 4.3.3. Continuous variables were summarized using mean and standard deviation, factor variables were summarized as counts and proportions.

With the vendor-specific, predefined time frames the calculated CPTT fall into one of three groups: 0/10/20 seconds. As time between 0 and 5 s would be categorized as 0, an impossible CPTT, we chose the mean value between 0 and 5 (2.5 s). Then, CPTT values were normalized according to the heart rate. This was achieved by dividing the CPTT by the duration of the RR-interval (NCPTT = CPTT / RR-interval), which can be roughly interpreted as the number of cardiac cycles needed for blood to circulate between the right and left ventricular cavities. Notably, previous studies have shown that normalizing CPTT provides a more reliable indicator of cardiac function, as it accounts for variations in heart rate [9].

### Statistical modeling

We conducted a simple linear regression with EF as the dependent variable and rest NCPTT as the independent variable. Normality and homoscedasticity of residuals were assessed using the DHARMa package. All statistical analyses described employed a p-value threshold significance at *p* < 0.05.

For the prediction study, an additional simple linear regression was performed. In this model, EF_Echo_ was the response variable, and EF_PET_ was the independent variable. Predictive performance was tested by leave-one-out-cross-validation (LOOCV), and the root mean squared error (RMSE) between the predicted EF and the true EF_Echo_ was computed.

## Results

### Patient characteristics

44 patients fulfilled all inclusion criteria and were selected for further analysis (Fig. 1). Demographic data of the patients are illustrated in Table 1. Age, BMI, weight, height and injected radioactivity were not gender dependent.

**Table 1:** Patient characteristics (means ± standard deviations, BMI = Body Mass Index, MBq = Megabecquerel)

Gender	Male: 28 (63.6%), Female: 16 (36.4%)
age (years)	72.4 ± 11.3
BMI (kg/m²)	27.5 ± 5.4
weight (kg)	81.3 ± 18.5
height (cm)	171.7 ± 8.5
[^82^Rb]RbCl (MBq)	595 ± 151.9

### Imaging parameters

As expected, CPTT values at rest are higher, compared to stress. This difference is corrected when CPTT is normalized to heart rate (Table 2A). Table 2B summarized the heart rate and blood pressure during PET imaging. The parameters derived from echocardiography can be found in Table 2C, including the completeness of the datasets.

**Table 2A:** CPTT and NCPTT values derived from dynamic PET imaging (means ± standard deviations)

CPTT_rest_ (seconds)	9.2 ± 4.2
CPTT_stress_ (seconds)	7.4 ± 3.6
NCPTT_rest_	10.4 ± 4.7
NCPTT_stress_	10.9 ± 5.4
EF_PET_ (%)	51.5 ± 13.5

**Table 2B:** Cardiac parameters during PET imaging (means ± standard deviations, HR = heartrate, systolic BP = systolic blood pressure)

HR_rest_ (b.p.m.)	70.1 ± 11.5
HR_stress_ (b.p.m.)	89.7 ± 13.8
systolic BP (mmHg)	135.2 ± 23.2

**Table 2C:** Parameters derived from echocardiography (means ± standard deviations)

		Missing (*n*, %)
EF_Echo_ (%)	56.4 ± 9	6, 14%
TAPSE (mm)	22.8 ± 4.5	15, 34%
E/A	0.9 ± 0.5	20, 45%

### NCPTT in relation to echocardiographic parameters

NCPTT_rest_, measured during [^82^Rb]RbCl-PET/CT, showed a mean of 10.4 ± 4.7 beats per cardiac cycle, while NCPTT_stress_ showed a mean of 10.9 ± 5.4 beats per cardiac cycle. Regarding left ventricular function, the mean of EF_PET_ was 51.5% ± 13.5 among all 44 patients, and the mean of EF_Echo_ was 56.4% ± 9 among 38 patients. Analysis of other measured echocardiographic parameters, such as left ventricular filling pressures, revealed a mean mitral valve E/A ratio of 0.9 ± 0.5 in 24 patients. Additionally, TAPSE, an indicator of systolic right ventricular function, showed a mean value of 22.8 ± 4.5 mm in 29 patients. We identified EF_Echo_ to be negatively associated with NCPTT_rest_ (beta = -0.77; CI: -1.32, -0.22; *p* = 0.007). All other independent variables were removed by the model selection process.

### Prediction of EFEcho

**Fig. 4:**
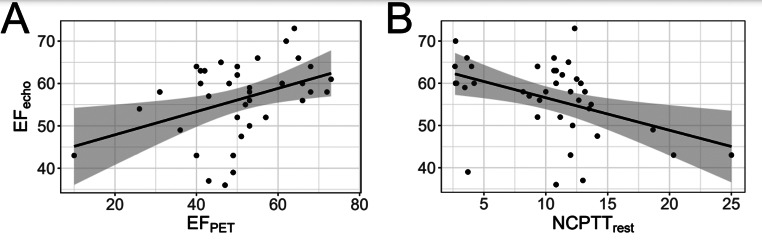
Scatter plots for EF_echo_ and EF_PET_ (A) and NCPTT (B). The univariate regression line is added for orientation

Given the identified association of EF_Echo_ and NCPTT_rest_, the predictive performance of NCPTT_rest_ for EF_Echo_ was investigated and compared to predictions of the EF_PET_ values derived from the gated [^82^Rb]RbCl-PET/CT analysis. Univariate correlation coefficients were 0.4 (*p* = 0.01) for EF_PET_ (Fig. 4A) and − 0.43 (*p* < 0.01) for NCPTT_rest_ (Fig. 4B). For the univariate predictive models for EF_Echo_, RMSE was 6.8% for the EF_PET_, and 6.0% for NCPTT_rest_, which indicates a slightly higher predictive performance for the NCPTT_rest_ model with a lower error.

## Discussion

In this retrospective study of patients referred for [^82^Rb]RbCl-PET/CT, we demonstrated a significant negative correlation between normalized cardiopulmonary transit time during rest (NCPTT_rest_), with the EF_Echo_ measured through TTE. Furthermore, we showed that NCPTT_rest_ had a slightly higher predictive performance for EF_Echo_, compared to the predictive performance of EF_PET_ with EF_Echo_.

CPTT is a parameter in cardiac imaging with increasing interest and acceptance. The measurement of CPTT using PET has emerged as a valuable non-invasive technique in the assessment of cardiovascular function, particularly in relation to LVEF [8]. The correlation between CPTT and LVEF has been extensively studied for CT and MRI, revealing consistent findings across various research [4–7, 10, 11]. Houard et al. conducted a prospective MRI study including 410 patients with heart failure with reduced ejection fraction (HFrEF) characterized by a LVEF of less than 35%. The study revealed a significant negative correlation between CPTT and LVEF, as well as further negative correlations between CPTT and right ventricular function, as measured by TAPSE. Additionally, they also demonstrated positive correlations between CPTT and the E/A ratio, a parameter representing echocardiographic filling pressures [7]. Another study, including patients with heart failure with preserved and reduced ejection fraction (HFpEF, HFrEF), demonstrated a correlation between global central transit time, a parameter corresponding to CPTT, and cardiac indices such as LVEF and right ventricular ejection fraction (RVEF), with the correlation being particularly strong in HFrEF patients [10]. These findings emphasize the influence of systolic dysfunction on transit time prolongation. Also, according to Harms et al., CPTT was significantly correlated with LVEF (*r*=-0.513,*p* < 0.001). Interestingly, the negative correlation between CPTT and LVEF is also present in our collective with outpatients without significant heart failure (0 patients with EF_Echo_ <35% and 4 patients with EF_PET_ <35%). CPTT during rest and stress were highly correlated (*r* = 0.842,*p* < 0.001), which led to presenting results only for CPTT during rest. Their model furthermore suggests that there is no significant influence derived from CPTT during stress [12]. Echocardiography was chosen due to its widespread use and availability, even tough EF_PET_ correlates better with EF_MRI_ using cardiac MRI.

Our findings suggest that NCPTT could potentially serve as a more reliable parameter for the prediction of EF_Echo_ and therefore the cardiac function in comparison to EF_PET_ measured in PET/CT. This is the first time that this has been shown with a predictive model. These findings underline previous findings demonstrating that PET- or SPECT-derived EF is somewhat prone to error [2, 3]. This is especially true, when it comes to differences in software [13]. Here, correlation coefficients for LVEF interobserver mean rest ranged from 0.68 to 0.89. Furthermore, quantification of cardiac function highly depends on imaging reconstruction [14]. On the other hand, a study has shown that [^82^Rb]RbCl-PET/CT displays a high reproducibility between scans when measuring different parameters, such as LVEF [15]. Furthermore, earlier studies also demonstrated that EF_PET_ between 18 and 59% were highly correlated to values derived from TTE (*r* = 0.97,*p* = 0.01) [16]. Similarly, another study showed that LVEF has a good correlation between resting TTE and PET MPI at stress (*r* = 0.83,*p* < 0.001) and rest (*r* = 0.80,*p* < 0.001) [17]. Of note, our data was acquired under clinical, real-life conditions with potential higher deviations compared to controlled reading studies under laboratory conditions as shown in our patient presentation.

Additionally, while our findings have not found any sex-specific differences in CPTT, previous literature has described male patients having significantly longer CPTT than female patients [11, 18]. When comparing the CPTT during stress with the CPTT at rest, one can see that the time it takes for the blood to travel from the left to the right heart shortens. However, when looking at the NCPTT, the time is even slightly prolonged during stress compared to rest. This can be explained by the fact that a higher heart rate results in a smaller RR interval. Consequently, this leads to higher normalized values when you revisit the formula (NCPTT = CPTT / RR-interval).

The 10-second framesets during [^82^Rb]RbCl-PET/CT were chosen according to vendor-supplied defaults. These frames were longer in comparison to other studies [18], potentially increasing the likelihood of image artifacts during the reconstruction process.

CPTT is a reliable parameter that not only reflects ventricular function, but when prolonged, has also been associated with various pathological conditions such as pulmonary hypertension, COPD and congestion such as HFpEF and HFrEF [4, 5, 12]. Detecting subclinical signs of reduced heart function can be difficult. Therefore, finding a way to detect them as early as possible, is of extreme importance in order to prevent further progression of disease. These findings emphasize the importance of CPTT as a potentially valuable parameter in the assessment and management of cardiovascular diseases and prediction of adverse cardiovascular outcomes [11]. A key advantage of CPTT measurement during [^82^Rb]RbCl-PET/CT is that it can be obtained without requiring additional imaging time or radioactive tracer application. Yet, it provides interesting additional diagnostic information regarding cardiovascular and pulmonary function.

### Limitations

A limitation of this paper is the predefined 10-second timeframe in the first two minutes of [^82^Rb]RbCl-PET/CT examination, which demand statistical assumptions, such as estimating and replacing a CPTT of 0 s with 2.5 s. Longer frames could also lead to potential image artifacts during reconstruction, which could be reduced by choosing shorter framesets. In addition, the 100-day interval between the PET and echocardiographic examination is extensive, as the echocardiographic dataset may have undergone significant changes during this period. Another important limitation is the incomplete echocardiographic data set, which limits the reliability of comparisons and conclusions. Finally, while we have included data on age, gender, BMI, and LVEF, information on cardiovascular risk factors and heart failure classification (HFrEF or HFpEF) was not systematically recorded in this retrospective analysis.

## Conclusions

In conclusion, CPTT is a non-invasive marker of cardiopulmonary circulation that is straightforward to measure in routine [^82^Rb]RbCl-PET/CT. Given its negative correlation with LVEF derived from TTE and the higher predictive power, this could be an interesting additional marker in the assessment of left ventricular function.

## Data Availability

The data presented in this study are available upon request from the corresponding author. The data are not publicly available because they contain information that could compromise research participant privacy.

## Abbreviations

[^82^Rb]RbCl: Rubidium-chloride
BMI: Body mass index
CAD: Coronary artery disease
CMD: Coronary microvascular dysfunction
cMRI: Cardiac magnetic resonance imaging
COPD: Chronic obstructive pulmonary disease
CPTT: Cardiopulmonary transit time
E/A: E-Wave to A-Wave ratio
EANM: European Association of Nuclear Medicine
EF: Ejection fraction
EF_Echo_: Left ventricular ejection fraction derived from TTE
EF_PET_: Left ventricular ejection fraction derived from PET
HFpEF: Heart failure with preserved ejection fraction
HFrEF: Heart failure with reduced ejection fraction
Kcps: Kilocounts per second
LOOCV: Leave-one-out-cross-validation
LV: Left ventricle
LVEF: Ejection fraction of the left ventricle
MBF: Myocardial blood flow
MBq: Megabecquerel
mcg: Micrograms
MFR: Myocardial flow reserve
mL: Milliliters
mmHg: Millimeters of mercury
MPI: Myocardial perfusion imaging
NCPTT: Normalized cardiopulmonary transit time
NCPTT_rest_: Normalized cardiopulmonary transit time during rest
NCPTT_stress_: Normalized cardiopulmonary transit time during stress
OSEM: Ordered-subset expectation maximization
PET/CT: Positron Emission Tomography/Computer Tomography
RMSE: Root mean squared error
RVEF: Ejection fraction of the right ventricle
SPECT: Single photon emission computed tomography
SUV: Standardized uptake values
TAPSE: Tricuspid annular plane systolic excursion
TOF: Time of flight
TTE: Transthoracic echocardiography
VOI: Volume of interest
